# A Proposal for Formation of Archaean Stromatolites before the Advent of Oxygenic Photosynthesis

**DOI:** 10.3389/fmicb.2016.01784

**Published:** 2016-11-15

**Authors:** John F. Allen

**Affiliations:** Research Department of Genetics, Evolution and Environment, University College LondonLondon, UK

**Keywords:** cyanobacteria, redox regulation, Great Oxidation Event, boring billion, protoerozoic, protocyanobacterium, redox switch hypothesis, two-component regulatory systems

## Abstract

Stromatolites are solid, laminar structures of biological origin. Living examples are sparsely distributed and formed by cyanobacteria, which are oxygenic phototrophs. However, stromatolites were abundant between 3.4 and 2.4 Gyr, prior to the advent of cyanobacteria and oxygenic photosynthesis. Here I propose that many Archaean stromatolites were seeded at points of efflux of hydrogen sulfide from hydrothermal fields into shallow water, while their laminar composition arose from alternating modes of strictly anoxygenic photosynthetic metabolism. These changes were a redox regulatory response of gene expression to changing hydrogen sulfide concentration, which fluctuated with intermittent dilution by tidal action or by rainfall into surface waters. The proposed redox switch between modes of metabolism deposited sequential microbial mats. These mats gave rise to alternating carbonate sediments predicted to retain evidence of their origin in differing ratios of isotopes of carbon and sulfur and in organic content. The mats may have arisen either by replacement of microbial populations or by continuous lineages of protocyanobacteria in which a redox genetic switch selected between Types I and II photosynthetic reaction centers, and thus between photolithoautotrophic and photoorganoheterotrophic metabolism. In the latter case, and by 2.4 Gyr at the latest, a mutation had disabled the redox genetic switch to give simultaneous constitutive expression of both Types I and II reaction centers, and thus to the ability to extract electrons from manganese and then water. By this simple step, the first cyanobacterium had the dramatic advantage of emancipation from limiting supplies of inorganic electron donors, produced free molecular oxygen as a waste product, and initiated the Great Oxidation Event in Earth’s history at the transition from the Archaean to the Paleoproterozoic.

## The Problem: Stromatolites A Billion Years Before the Cyanobacteria that Make Them

**FIGURE 1:**
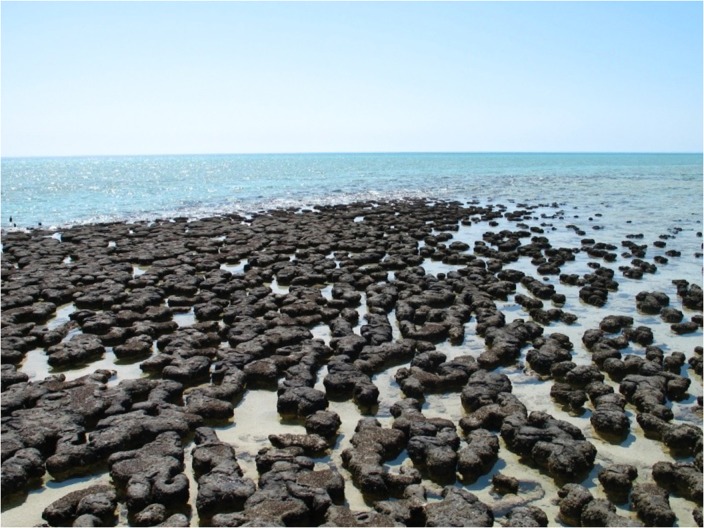
**Stromatolites at Hamelin Pool, Western Australia, 4 October 2007.** Photograph kindly provided by Dr. Catherine Colas des Francs-Small.

Stromatolites today are unusual features of shorelines. They grow typically in anoxic, shallow, hypersaline waters (**Figure 1**) and are constructed by microbial communities whose primary producers are cyanobacteria. In the pre-Cambrian Palaeozoic and Archaean eons, stromatolites were much more abundant than today, took on a wide variety of sizes and morphologies, and built massive reefs of sediments whose organic and inorganic carbon isotopic signatures suggest large-scale solar energy conversion with primary productivity comparable with that seen today in coral reefs (Bosak et al., 2013).

Archaean stromatolites are an anomaly. If cyanobacteria had yet to appear, what made them? There is clear and multiple, independent lines of evidence that the Earth’s oceans and atmosphere were anoxic for roughly half its history, that is, until 2.4 Gyr (Knoll et al., 2016). Oxygen-evolving photosynthesis then emerged from anoxygenic photosynthesis with the advent of cyanobacteria – the only oxygenic phototrophic prokaryotes (Schopf, 2011). Nevertheless stromatolites comprise exposed sediments, notably in South Africa and Western Australia, that are dated unambiguously up to at least a billion years before the start of the rise in atmospheric oxygen concentration (Hofmann et al., 1999; Allwood et al., 2006; Nutman et al., 2016). It has been considered that cyanobacteria may have first emerged during the early or mid-Archaean, giving “whiffs of oxygen” (Holland, 2006; Summons et al., 2006). However, the abundance of Archaean stromatolite coastal reefs (Schopf et al., 2007; Bosak et al., 2013) is likely to reflect abundant photosynthetic microbial activity that could be expected to have created a much earlier Great Oxidation Event if cyanobacteria alone had been responsible.

## Hypothesis: Laminar Microbial Mats from Alternating Modes of Metabolism

**FIGURE 2:**
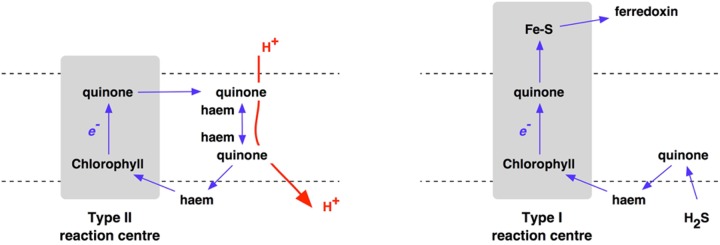
**Electron transport chains incorporating either Type II **(left)** or Type I **(right)** reaction centers of anoxygenic photosynthesis**.

**FIGURE 3:**
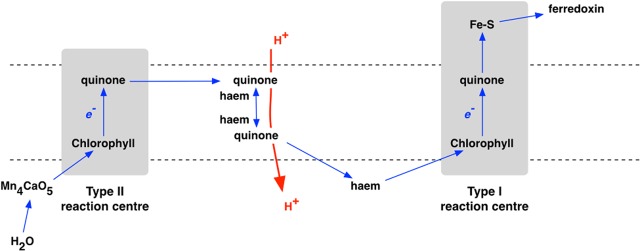
**The electron transport chain of oxygenic photosynthesis, where Types II and I reaction centers are linked, in series, as photosystem II (PS II) and photosystem I (PS I), respectively**.

Oxygenic photosynthesis appeared with the first cyanobacterium (Fischer et al., 2016). Photosynthetic oxygen production always requires two, connected photosystems (Nelson and Junge, 2015). Photosystem I uses light energy at its primary photochemical reaction center to oxidize a chlorophyll molecule that donates its electron to a series of iron–sulfur proteins (Fromme et al., 2001; Amunts et al., 2007), and then on to coupled assimilatory metabolism such as the Benson-Calvin cycle of CO_2_ fixation. In contrast, the oxidized chlorophyll of the photosystem II reaction center passes its electron to a pair of quinone molecules (Nitschke and Rutherford, 1991; Brinkert et al., 2016). The photooxidised chlorophyll of photosystem II is reduced by electrons from water (Umena et al., 2011; Shen, 2015; Ho et al., 2016). Oxidation of two water molecules by transfer of four electrons releases one molecule of oxygen. Photosystem I and photosystem II have homologs in the single, separate photosystems of anoxygenic photosynthetic bacteria, each with either a Type I or a Type II photochemical reaction center (**Figure 2**), while it is clear that Types I and reaction centers are themselves homologous (Schubert et al., 1998) – related by descent from a common ancestor. For the origin of oxygenic photosynthesis, the reaction centers of two independent photosystems must have become connected, electrically in series, to allow two separate light reactions (Hill and Bendall, 1960) to transfer electrons from water to the iron–sulfur acceptors that supply electrons at low redox potentials to assimilatory reactions (Hohmann-Marriott and Blankenship, 2011) (**Figure 3**).

One proposal for the way in which this close coupling began is that genes for each of the two reaction centers, Types I and II, were present as alternatives in a versatile, anoxygenic photosynthetic bacterium termed the “protocyanobacterium” (Allen, 2005; Allen and Martin, 2007).

Photosynthesis is a light-driven redox reaction that can be supported by any of a variety of electron donors (sources) and acceptors (sinks). Archaean electron donors included hydrogen, ferrous iron, and hydrogen sulfide, each varying in availability with time and location (Bosak et al., 2013). Major Archean electron acceptors were carbon dioxide and nitrogen gas, as today, but not oxygen.

The overall process of photosynthesis as light-driven electron or hydrogen transfer to CO_2_ is summarized in the van Niel equation (1) (Van Niel, 1954):

$${2H}_{2}A+{CO}_{2}\overset{light}{\underset{{4e}^{-}}{\to }}{CH}_{2}O+H_{2}+2A$$

Where H_2_A is an electron donor, A is its oxidation product, and CH_2_O represents sugars, organic products of CO_2_ fixation. H_2_A can be inorganic, giving photolithotrophy, or organic, giving photoorganotrophy. Oxygenic photosynthesis is the special case of photolithotrophy where H_2_A is H_2_O and 2A is O_2_. Anoxygenic photosynthesis today typically relies either on inorganic donors, as seen in green and purple sulfur bacteria, which are obligate anaerobes, or on organic donors, as seen in green and purple non-sulfur bacteria, which today can often tolerate oxygen, but not while remaining phototrophic.

It should be noted that CO_2_ is an assimilatory substrate and not a direct electron acceptor for photosynthesis, and the van Niel equation (1) can be regarded as the sum of two half reactions, (2) and (3):

$${2H}_{2}A\overset{light}{\to }{4e}^{-}+{4H}^{+}+2A$$

$${CO}_{2}+{4e}^{-}+{4H}^{+}\to {CH}_{2}O+H_{2}O$$

Together with coupled synthesis of ATP, photosynthetic reduction of ferredoxin drives eventual assimilation of CO_2_ and N_2_.

N_2_ fixation, reaction (4), is a “dark reaction” analogous to reaction (3):

$$N_{2}+{8e}^{-}+{8H}^{+}\to {2NH}_{3}+H_{2}$$

The overall equation for photosynthetic N_2_ fixation is then the sum of (2) and (4), giving (5):

$${4H}_{2}A+N_{2}\overset{light}{\underset{{8e}^{-}}{\to }}{2NH}_{3}+H_{2}$$

I suggest that, in addition to CO_2_ fixation, light-driven N_2_ fixation with an unlimited supply of reductant may have been an immediate benefit of the use of water as an electron donor by the first cyanobacterium. Phylogenetic analysis suggests that the earliest cyanobacteria were indeed filamentous nitrogen-fixers (Dagan et al., 2013). While inhibition of nitrogen fixation by O_2_ would have been an immediate consequence of water oxidation, this penalty could have been offset by dilution of free O_2_, into the anoxic environment. Environmental O_2_ concentration may then have risen slowly, eventually to reach a steady state where a low maximal rate of nitrogen fixation sustained a correspondingly low rate of photosynthetic water oxidation. An atmospheric oxygen concentration of about 10% of that seen today is a maximum beyond which nitrogen fixation cannot proceed (Berman-Frank et al., 2003; Falkowski, 2007). It is possible to imagine that atmospheric oxygen was maintained by this dynamic equilibrium at about 10% of its present concentration during the “boring billion” (Holland, 2006; Johnston et al., 2009; Lyons et al., 2014) of the Proterozoic.

Major differences between oxygenic and anoxygenic photosynthesis are listed in **Table 1**.

**Table 1:** Characteristics of oxygenic and anoxygenic photosynthesis.

Oxygenic photosynthesis	Anoxygenic photosynthesis
Light-driven transmembrane electron transfer	Light-driven transmembrane electron transfer
Coupled to proton translocation	Coupled to proton translocation
Including a proton-motive Q-cycle through a cytochrome *b-f* complex	Including a proton-motive Q-cycle through a cytochrome *b-c*_1_ complex
Two photosystems or “light reactions”: Type I (PS I) and Type II (PS II)	One photosystem or “light reaction” of either Type I or Type II.
Includes non-cyclic electron transport pathway with H_2_O as the initial electron donor	Non-cyclic electron transport pathway with inorganic electron donors (e.g., H_2_S, Fe^2+^, H_2_) or organic electron donors (e.g., succinate, acetate, and pyruvate).
Special case of the van Niel equation	Other special cases of the van Niel equation
Carbon dioxide fixation by the Benson–Calvin pathway (a.k.a. reductive pentose phosphate pathway)	Carbon dioxide fixation by the Benson–Calvin pathway (a.k.a. reductive pentose phosphate pathway) OR by other pathways such as the “reverse” (i.e., reductive) TriCarboxylic Acid cycle
Makes oxygen	Inhibited by oxygen
In cyanobacteria and chloroplasts	In purple and green photosynthetic bacteria, and heliobacteria
Resulted in the Great Oxidation (or Event; oxygen-rich atmosphere and eventually oceans; aerobic respiration; ozone layer and life on land; end of MIFS and BIFS from Fe^2+^→Fe^3+^; N as nitrite/nitrate; S as sulfide/sulfate; eukaryotes; multicellularity	Resulted in increased biomass in coastal microbial mats and stromatolites as free energy input from sunlight added to geochemical sources.
Appeared at the Archaean to (paleo)proterozoic boundary ∼2.5 Gyr (or earlier if “whiffs of O_2_” are real and a signature)	Appeared early in the Archaean eon from 3.8 Gyr


Anaerobic, anoxygenic photosynthesis is likely to have been be the only option for Archaean primary production using the energy of sunlight. Where H_2_S is available as an electron donor, Type I photosynthetic reaction centers transport electrons to iron–sulfur electron acceptors – ferredoxins – in a linear or non-cyclic electron transport pathway. In contrast, where organic electron donors such as succinate or pyruvate are used, Type II photosynthetic reaction centers use quinone acceptors that pass electrons back to the reaction center, giving proton-motive cyclic electron flow and coupled ATP synthesis **Figure 2**.

Hydrothermal activity would have produced marked variation in concentration of dissolved inorganic electron donors in Archaean euphotic zones such as littoral, coastal, and river environments where microbial mats were formed (Homann et al., 2015). Environments replete with ferrous iron or hydrogen sulfide would have changed repeatedly and reversibly to ones where the only available electron donors were organic – these locations would therefore have alternated in their provision for photosynthesis by Types I and II reaction centers.

**FIGURE 4:**
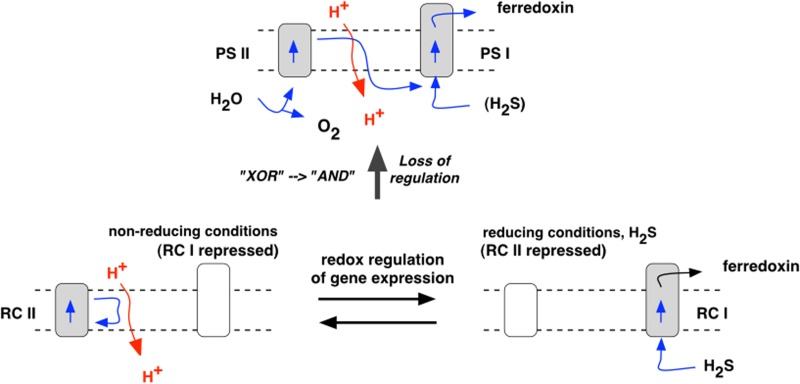
**The hypothetical protocyanobacterium.** Expression of Type I center genes in the presence of H_2_S gives a Type I reaction center (RC I) and is accompanied by repression of Type II genes. In the absence of H_2_S, Type II genes are induced, giving a Type II reaction center (RC II), and Type I genes become repressed. Subsequent impairment of redox regulatory control allows expression and co-existence of both Type I and Type II reaction centers, with complementary functions. In place of H_2_S, the Type II center, as PS II, oxidizes water, liberating oxygen, and donates electrons to the Type I center, as PS I. The proposed loss of the redox regulatory switch replaces the logical (Boolean) relation “Type I ***XOR*** Type II” (each type excluding the other) with “Type I ***OR*** Type II” (either is, and both are, allowed). This in turn leads to “Type I ***AND*** Type II” when interdependency of PSs I and II is established in the non-cyclic electron transport chain of oxygenic photosynthesis.

A versatile, anoxygenic bacterium with inducible Types I and II photochemical reaction centers has been proposed as the immediate forerunner of oxygenic cyanobacteria (Allen, 2005) (**Figure 4**). Such a protocyanobacterium would have had a minimum quantum requirement for four-electron transport of 4, which conferred, at limiting light intensities, an energetic advantage over oxygenic photosynthesis with its equivalent quantum requirement of at least 8. This advantage suggests that protocyanobacteria may persist today, albeit at low abundance in specialized environments where light is a limiting factor for growth and there is a fluctuating availability of inorganic and organic electron donors. In the late Archaean, however, CO_2_ concentration was much higher than it is today, solar luminosity was lower, and facultative Type I–Type II photosynthesis can be envisaged as an optimal growth strategy where supplies of an inorganic electron donor varied. One reason for this variation may have been the cyclic depletion of Fe^2+^ when insoluble Fe^3+^ salts were formed, thus giving rise to the banded iron formations seen throughout the Archaean and well into the Proterozoic. Fe^2+^ depletion could also have arisen where H_2_S was in excess of Fe, giving FeS and FeS_2_ (pyrite). However, when Fe^2+^ ran out, and, in any case, in shallow waters above hydrothermal fields, hydrogen sulfide derived ultimately from magma would have been introduced at specific points, and along fissures, in the bedrock, and would have been available an electron donor both by sulfur bacteria and by the protocyanobacteria. The competitive advantage of the protocyanobacteria would have been maintenance of populations of cells with their core metabolisms and genetic systems always in place. It is thus possible that protocyanobacteria contributed to the massive stromatolite and microbialite reefs laid down before, after, and across the Archaean-Proterozoic boundary.

**FIGURE 5:**
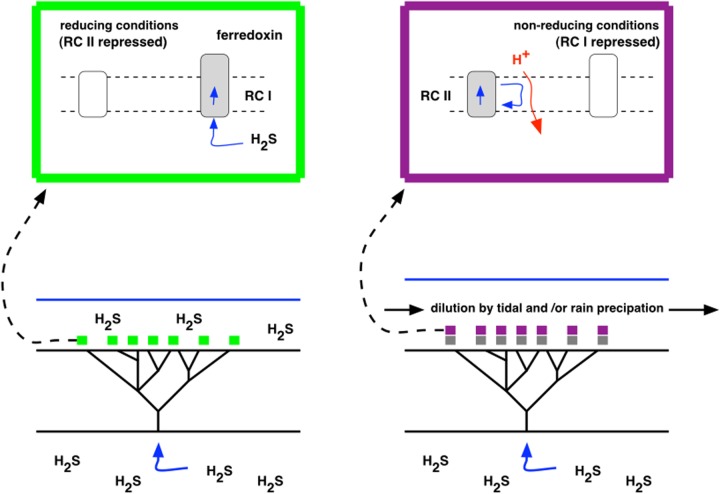
**A model for seeding of stromatolites.** **Left** (green box): H_2_S is exhaled from a hydrothermal vent or fissure, accumulates, and serves as an electron donor to Type I photosynthesis in photoautotrophic growth. **Right** (purple box): H_2_S is depleted by dilution through tidal action or rainfall, leaving Type II, photoheterotrophic growth, using organic compounds (gray) that accumulated as products of photosynthesis during the previous, photoautotrophic stage.

**FIGURE 6:**
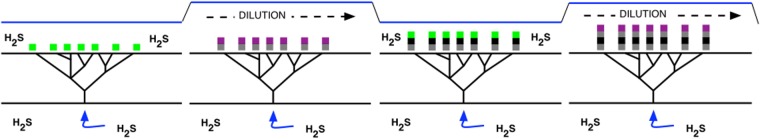
**A model for formation and growth of stromatolites.** Sequential concentration and dilution of hydrothermally derived H_2_S results in alternation of Types I and II photosynthesis, and in alternation of photoautotrophy and photoheterotrophy. Sequential deposition of layers of cellular material from each mode of metabolism produces a laminar substrate that raises the growing microbial mat incrementally nearer to the surface of the water and source of light.

Observations on the Strelley Pool formation of the Pilbara Craton in Eastern Australia shows that stromatolites were abundant at 3.4 Gyr (Wacey, 2010), and their distribution coincides spatially with hydrothermal venting as judged by lithography and with the presence of sulfur isotopes in both organic (kerogen) and inorganic (carbonate) stromatolite composition (Sugitani et al., 2015). The proposed process of stromatolite building as H_2_S supply fluctuated is outlined schematically in **Figure 5**. **Figure 6** provides a context for hydrothermally derived columnar stromatolites built by influx of H_2_S.

## Predictions of the Hypothesis

### 1. Location of Stromatolites

In contrast to the first bacteria and archaea, and to microbiota growing in the vicinity of submarine hydrothermal vents, all of which were chemoautotrophic, by the Archaean extensive microbial growth had become dependent on photosynthesis, and required sunlight. Depending on solutes and turbidity, water transmits light only to limited depths at wavelengths and flux densities that can be used by photosynthesis (Falkowski and Raven, 2007). Thus stromatolites grew in shallow water, and became abundant where large surface areas were exposed to sunlight, typically adjacent to shorelines or in river estuaries.

### 2. Coincidence of Individual Stromatolites with Hydrothermal Outlets

Where H_2_S was the photosynthetic electron donor, photolithoautotrophic growth would have been favored at precise locations where H_2_S flux gave rise to optimal steady-state concentrations, that is, in the vicinity of sources of H_2_S from hydrothermal activity. Such coastal hydrothermal fields supported microbial communities and stromatolite formation at 3.4 Gya as seen in the Strelley Pool Formation, Pilbara Craton, Western Australia (Sugitani et al., 2015). Fractured rock in hydrothermal fields is predicted to have given a specific spatial pattern of seeding or initiation of stromatolite growth, corresponding to the pattern of entry to dissolved H_2_S into the water. It is therefore proposed that each individual Archaean stromatolite column can be assigned to an individual channel or fissure in the field’s bedrock.

### 3. Delta ^13^C Variation with the Frequency of Stromatolite Laminae

Enzyme-catalyzed reactions discriminate in favor of lighter isotopes of elements in substrates. ^13^C is about one percent of total carbon on Earth, the rest being ^12^C. Photoautotrophic growth thus preferentially assimilates ^12^CO_2_ into organic products. The greater the number of enzymatic steps involved, the greater the depletion of ^13^C in the photosynthate, as illustrated today by the differing isotopic compositions of C_3_ and C_4_ crop plants (von Caemmerer et al., 2014). If stromatolite laminae were formed by alternating phases of autotrophic and heterotrophic growth, then the delta-^13^C composition of the organic material should fluctuate and be expected to be lowest in the heterotrophic layers, reliant on metabolic reactions additional to those of the preceding, primary carboxylation step of CO_2_ assimilation. Photoautotrophically obtained carbon in alternate laminae will still show a lower delta-^13^C than that of the surrounding environment. A clear pattern is also to be expected of increased ^13^C in carbonates precipitated from the surrounding medium. These would be richest in ^13^C where they had selectively been depleted of ^12^C by CO_2_ assimilation, while showing evidence of re-enrichment with ^12^C from the environment during the heterotrophic phase of growth.

### 4. Delta ^34^S Variation with the Frequency of Stromatolite Laminae

The same logic applies to predicted sulfur isotopic signatures, where negative relative values of the ratio ^34^S/^32^S are taken to indicate a biological origin. Large negative ^34^S/^32^S values are reported for bulk barite (barium sulfate) deposits in chert of the Dresser formation (Philippot et al., 2007). These values could be predicted to have arisen from an H_2_S-oxidizing, Type I photosynthesis, where the initial enzyme-catalyzed reaction is that of sulfide-quinone oxidoreductase (Arieli et al., 1994). If the biological mass fractionation of sulfur isotopes arose from alternating photolithotrophy and photoautotrophy, giving stromatolite laminae by a process as depicted in **Figures 5** and **6**, then, given sufficient spatial resolution, negative delta ^34^S excursions in products of lithotrophic growth should be coincident with negative delta ^12^C excursions reporting autotrophy.

For both predictions 3 and 4, above, mass spectroscopy of Archaean stromatolite laminae nanometer-scale resolution (Lepot et al., 2008) may be required. Laminae can be as thin as a millimeter (Bosak et al., 2009), arguing for relatively transient deposition of sequential layers of microbial biomass.

## The Hypothesis Extended to Protocyanobacteria and the Redox Switch Hypothesis for the Origin of Oxygenic Photosynthesis

Each of the predicted observations 1–4, above, might be explained by cyclical replacement of anoxygenic microbial mats each composed of either sulfur (Type I) or non-sulfur (Type II) photosynthetic bacteria. However, another possibility is that one type of protocyanobacterium predominated and left signatures of alternating modes of metabolism supported by one genome containing genes for both Types I and II reaction centers. The advantage of this special case of the hypothesis is that it allows us to envisage simple steps from anoxygenic to oxygenic photosynthesis, a profound evolutionary transition otherwise lacking clear explanation supported by observation. The late Archaean abundance of the protocyanobacterium and its proposed role in the onset of the GOE makes further predictions 5–7, as follows.

### 5. Manganese Oxidation and Deposition As Precondition to the Emergence of the First Water-Splitting Enzyme Liberating Oxygen and Feeding Electrons to Photosystem II

Photosystem II abstracts four electrons from two water molecules, yielding one oxygen molecule. It does this by accumulating four positive changes in an inorganic prosthetic group of four manganese atoms, one calcium, and five oxygen atoms (Umena et al., 2011). A Type II reaction center oxidizing manganese seems likely to have been a precursor of the oxygen-evolving photosystem II reaction center (Khorobrykh et al., 2013) and might itself have evolved from a Type II reaction center capable of oxidizing chlorophyll *a* to chlorophyll *f* (Ho et al., 2016). A sequence of manganese carbonate from the Koegas Subgroup, Kaapvaal Craton, South Africa is reported at 2.41 Gyr, when free oxygen is reported to have been absent (Johnson et al., 2013). The bacterium that deposited manganese may have done so as a result of the first coupling of Types II and I reaction centers, and only later could higher oxidation states of manganese become re-reduced by water (Allen, 2014; Fischer et al., 2016). When this happened, environmental manganese would have ceases to be a substrate for lithotrophy, and manganese became obligatory as a catalyst of water oxidation, to be sequestered and conserved. It is possible that minerals such as hollandite with a unit cell resembling the water-oxidizing cluster of photosystem II accumulated from such biological concentration and deposition (Russell et al., 2008).

### 6. Persistence of Living Protocyanobacteria with Genes for Types I and II Reactions Centers

The redox switch hypothesis (Allen, 2005) rests on the assumption of an anoxygenic phototroph as an immediate precursor of the first true two-light reaction phototroph, whether the latter oxidized environmental manganese directly, or water by means of a derived manganese catalyst (Allen and Martin, 2007; Russell et al., 2008). It could be argued that the protocyanobacterium was out-competed and displaced by the cyanobacteria to which it gave rise, and eventually became extinct. There are, however, specialized environments today where a fluctuating supply of hydrogen sulfide into an anoxic, photic water column would confer a selective advantage upon growth by means of a single photosystem, where the quantum yield is twice that of growth requiring two light reactions. Facultatively oxygenic *Oscillatoria* species, including *Oscillatoria limnetica*, are today oxygenic in the absence of H_2_S but switch in the presence of H_2_S to a Type I-only photosynthesis (Oren and Padan, 1978). If an *Oscillatoria* species were to retain the capacity for photoheterotrophic growth using a photosystem II depleted of water-oxidizing complex then its quantum requirement (of four) would be lower in both of its modes metabolism than a that (of eight) of a regular cyanobacterium. The protocyanobacterium would be better adapted than cyanobacteria to growth at low light intensity. It seems worth looking for Type I–Type II versatile phototrophs in anoxic, low-light environments with variable concentration of dissolved H_2_S. Stromatolite formation by anoxygenic photosynthesis may have continued through the Proterozoic (Johnston et al., 2009), gradually becoming displaced by facultative, oxygenic photosynthesis in cyanobacteria resembling extant *Oscillatoria* species.

### 7. Persistence of Living Protocyanobacteria with a Recognizable Redox Genetic Switch

Cyanobacteria (Ashby and Houmard, 2006) and bacteria in general (Stock et al., 2000) respond to environmental change by means of two-component regulatory systems. These systems consist of a histidine sensor kinase that transfers a phosphoryl group to a response regulator if, and only if, a specific environmental change occurs. The response regulator controls gene expression, usually though not exclusively at the level of transcription, in such a way as to bring about a change that better adapts the organism to its changed environment. Redox state of electron carriers responds rapidly to relevant environmental changes, and redox sensor kinases (Allen, 1993) are known to regulate photosynthetic gene transcription in cyanobacteria (Li and Sherman, 2000) and chloroplasts (Puthiyaveetil et al., 2008). A two-component redox genetic switch has been characterized that selects between transcription of photosystems I and II reaction center apoproteins in both chloroplasts (Pfannschmidt et al., 1999) and cyanobacteria (Ibrahim et al., 2016a). A protocyanobacterial version of this system, centered on the conserved Histidine Kinase 2 (Ibrahim et al., 2016b), could govern transcription of Types I and II reaction center genes and underlie the metabolic adaptability of the proposed protocyanobacterium. A simple mutation that impairs the switch could have been the beginning to two photosystems functioning simultaneously and in series (Allen, 2005, 2014).

## Discussion

Throughout Earth’s history, its crust, lithosphere, hydrosphere and atmosphere have undergone immense changes in overall composition (Nisbet and Sleep, 2001), while environments for life have been dynamic on a wide range of time scales (Allen, 1998). Life’s capacity to adapt is one of its defining features (Allen, 2010) making regulation at the boundary of internal and external environments coincident with mechanisms of nutrient exchange and energy transduction; a requirement for life’s origin (Mitchell, 1957; Sousa et al., 2013). By the Archaean the planet’s surface had separated into solid, liquid, and gaseous phases – into land and sea sharing a turbulent atmosphere. Tidal action, evaporation and precipitation would have created varying concentrations of solutes, notably at shore-lines and in rock pools, lakes, and rivers. When photosynthesis began to supply an input of free energy, itself varying diurnally and seasonally, then shallow waters, the euphotic zones, are most likely to have become locations of the greatest biological activity and primary productivity.

Here I suggest that alternating Types I and type II photosynthesis is a plausible mechanism for the seeding (**Figure 5**) and growth (**Figure 6**) of stromatolites, such as those recorded in Archaean stratigraphy, in the absence of cyanobacteria. It is further suggested that this type of versatile phototrophic growth occurred in single lineages of a protocyanobacterium that was a natural precursor of the first oxygenic cyanobacteria. The single step from Type I-OR-Type II photosynthesis to Type I-AND-Type II photosynthesis gave rise to two-light reactions, water-oxidizing, oxygenic photosynthesis (Allen, 2005; Allen and Martin, 2007; Russell et al., 2008), proliferation of genes for oxidation-reduction enzymes (Baymann et al., 2003; David and Alm, 2011), and to emancipation of biology from fleeting and potentially rate-limiting supplies of inorganic electron donors. The evolutionary success of this transition at 2.4 Gyr was the basis of the Great Oxidation Event, one of the greatest transitions in the evolution of life on our planet (Lyons et al., 2014).

## Author Contributions

The author confirms being the sole contributor of this work and approved it for publication.

## Conflict of Interest Statement

The authors declare that the research was conducted in the absence of any commercial or financial relationships that could be construed as a potential conflict of interest.
